# Down expression of *lnc-BMP1-1* decreases that of *Caveolin-1* is associated with the lung cancer susceptibility and cigarette smoking history

**DOI:** 10.18632/aging.102633

**Published:** 2020-01-04

**Authors:** Xiaoxuan Ling, Yinyan Li, Fuman Qiu, Xiaoxiao Lu, Lei Yang, Jinbin Chen, Tiegang Li, Di Wu, Huali Xiong, Wenpeng Su, Dongsheng Huang, Jiansong Chen, Binyao Yang, Hongjun Zhao, Chenli Xie, Yifeng Zhou, Jiachun Lu

**Affiliations:** 1 The State Key Lab of Respiratory Disease, The First Affiliated Hospital, Guangzhou Medical University, Xinzao, Guangzhou, China; 2 The School of Public Health, The Institute of Environmental and Health of Dongguan Key Laboratory, Guangdong Medical University, Dongguan, China; 3 The School of Public Health, The Institute for Chemical Carcinogenesis, Collaborative Innovation Center for Environmental Toxicity, Guangzhou Medical University, Guangzhou, China; 4 Department of English and American Studies, Faculty of Languages and Literatures, Ludwig Maximilian University (LMU), Munich, Germany; 5 Shenzhen Longhua District Central Hospital, Shenzhen, Guangdong, China; 6 The Fifth People’s Hospital of Dongguan, Dongguan, Guangdong, China; 7 Department of Genetics, Medical College of Soochow University, Suzhou, China; 8 Guangzhou Center for Disease Control and Prevention, Guangzhou, China

**Keywords:** *lnc-BMP1-1*, *Cav-1*, epigenetic modification, lung cancer, cigarette smoke

## Abstract

*Lnc-BMP1-1* is a lncRNA transcribed from *SFTPC* (surfactant associated protein C), a lung tissue specific gene encoding pulmonary-associated surfactant protein C (SPC) that is solely secreted by alveolar typeⅡ epithelial cells, among which the ones with *SFTPC+* might be transformed into lung adenocarcinoma cells. Caveolin-1 (*Cav-1*) is a candidate tumor suppressor gene and is vital for coping with oxidative stress induced by cigarette smoke. When comparing lung cancer tissues with their adjacent normal tissues, the expression of *lnc-BMP1-1* were decreased, especially in patients with cigarette smoking history (*P*=0.027), and positively associated with the expression of *Cav-1* (*P<*0.001). When comparing to A549 cells transfected with empty vector (A549-NC cells), the expression level of *Cav-1* in A549 cells with over-expressed *lnc-BMP1-1* (A549-BMP cells) was increased along with the decreased level of HDAC2 protein. The drug sensitivity of A549-BMP cells to Doxorubicin hydrochloride (DOX) was increased; the growth and migration capability of A549-BMP cells were inhibited along with the decreased protein level of Bcl-2 and DNMT3a; the growth of tumor in nude mice injected with A549-BMP cells were inhibited, too. Furthermore, the *lnc-BMP1-1* and *Cav-1* expression was also down-regulated in the human bronchial epithelial (16HBE) cells treated with cigarette smoke extract (CSE).

## INTRODUCTION

Long noncoding RNAs (lncRNAs) with lengths ranging from 200nt to 100 knt were termed as ‘transcribe noise’ decades ago. However, now they have been proven to be essential regulators of gene expression [1]. For example, the silence of X chromosome is also attributed to the activation of HDAC3 by lncRNA *Xist* [2]. *MALAT1* (Metastasis-associated lung adenocarcinoma transcript 1) and *HOTAIR* (*HOX* transcript anti-sense RNA) are well known lung cancer associated lncRNAs, with various functions in biological courses [3–5]. H3K27me3, the functional marker of *HOTAIR*, interacts directly with the epigenetically related molecular PRC2 (polycomb repressive complex2) [6]. However, it is widely accepted that the functions of lncRNAs and the detailed mechanisms remain largely unexplored [7], which might be key issues for cancer research [8].

Lung cancer (LC) has continued to threaten the lives of people worldwide, with a five-year survival rate remaining at a level of about 18% [9]. Along with the rising tobacco consumption in China, incidence rate of lung cancer is also increasing [10–12]. The acquired drug resistance in lung cancer therapy has shown that the exploring for biomarkers for therapy is too vital to be replaced [13], among which lncRNAs are promising [14–16]. For the confirmed correlation between cigarette smoke (CS) and lung cancer, we are interested in the role that lncRNAs play in CS induced lung carcinogenesis. One literature had revealed that cigarette smoke extract (CSE) could induce malignant transformation of human bronchial epithelial (HBE) cells through up-regulating the expression of *HOTAIR* by signal transducer and activator of transcription 3 (*STAT3*) to integrate inflammation with the epithelial-mesenchymal transition (EMT) [17]. Being correlated with smoking status, taurin-upregulated gene1 (*TUG1*) is significantly down-regulated in non-small cell lung cancer patients [18]. The siRNA knockdown of *SCAL1* (smoke and cancer-associated lncRNA-1) in HBE cells shows a significant potential of cytotoxicity induced by CSE in vitro, acting on downstream *Nrf2* (nuclear factor erythroid 2-related factor) to regulate gene expression and mediate oxidative stress protection in airway epithelial cells. These results further reinforce the involvement of lncRNAs in CS, mediating oxidative stress protection and lung cancer development [19].

*Cav-1* is considered as a candidate tumor suppressor gene [20] and an effective gene to cope with cigarette smoke deduced oxidative stress [21–23]. On the one hand, the survival is longer in cancer patients with higher *Cav-1* protein level. This stands true for patients of differet types of cancers, including gastric, lung, breast, colorectal cancer, and so on, by the representative mechanism of suppressing the *Ras/ MAPK*, *EGFR* pathway or activating the *p53* pathway. The protein level of *Cav-1* is positively correlated with nab-paclitaxel sensitivity of lung cancer patients because of its membrane protein characteristics favoring drug intake and transport [24–28]. On the other hand, in the stimuli of CSE, highly phosphorylated Cav-1 is strongly bound to *EGFR* so that the effectiveness of *EGFR-TKI* therapy is influenced [21–23]. With the stimuli of CSE, *Cav-1* also negatively regulates the autophagic label protein LC3B (autophagic protein microtubule-associated protein 1 light chain-3B) in COPD (chronic obstructive pulmonary disease) disease, thus protecting the epithelial cells from apoptosis and autophagy [29]. COPD is a risk factor for lung cancer [30, 31]. *Cav-1* encodes a 22 kDa protein and is located at chr7q31.1, a fragile genomic region also termed as FRA7G and is often deleted in cancers [32, 33]. A fragile genomic region might produce ncRNAs, among which the lncRNAs could recruit epigenetic modification complex to increase the stability of the region. Epigenetic modification of *Cav-1* is proved to be associated with lung cancer development [34]. DNA hyper-methylation or histone hypo-acetylation could suppress the expression of cigarette smoke-related genes and thus lead to pulmonary diseases [35–37]. Therefore, it’s possible for lncRNAs to influence the expression of *Cav-1* in way of epigenetic modification.

*Lnc-BMP1-1*, a lncRNA transcribed from DNA at chr8p21.3 (chr8: 22020592-22021052) in the intronic area of *SFTPC* (surfactant associated protein C), is 228nt in transcript length and contains two exons. The detailed information can be found on Lncipedia website (https://www.lncipedia.org/db/, NONCODE v4). *SFTPC* is a lung tissue-specific gene that encodes SPC protein, synthesized and secreted solely by alveolar typeⅡepithelial cells (AEC2s). Interestingly, the AEC2s with *SFTPC+* has the potential to be induced into lung adenocarcinoma cells [38], indicating the function of *SFTPC* in lung cancer development. The biological functions of *lnc-BMP1-1* have not yet been explored nor have been linked to any diseases thus far.

Based on the origin of *lnc-BMP1-1* and the potential function of *Cav-1* in conjunction with oxidative stress induced by cigarette smoke, we hypothesized that the decreased expression of *lnc-BMP1-1* may reduce the expression of *Cav-1* and lead to lung cancer. We analyzed the expression of *lnc-BMP1-1* and its relationship with clinical characteristics of lung cancer population, then tried to make clear the mechanism of *lnc-BMP1-1* regulating *Cav-1* through in vitro and in vivo experiments.

## RESULTS

### *Lnc-BMP1-1* is down-regulated in lung cancer patients and is associated with cigarette smoke history

**Figure 1:**
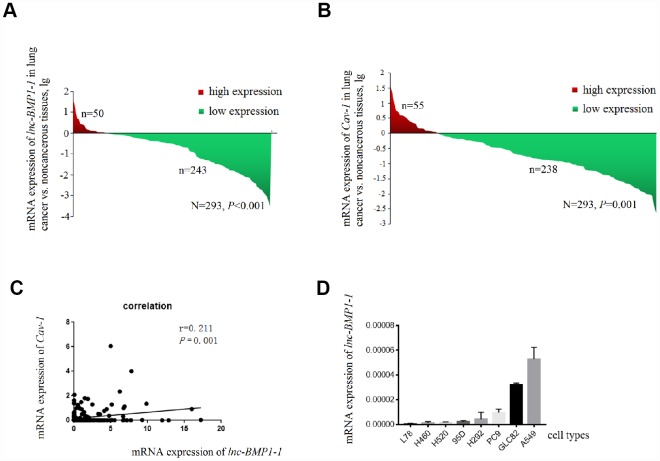
**The decrease of *lnc-BMP1-1, Cav-1* expression in lung cancer population and the expression of *Inc-BMP1-1* in lung cancer cell lines.** (**A**) The expression of *lnc-BMP1-1* was decreased in lung cancer patients; (**B**) The expression of *Cav-1* was decreased in lung cancer patients; (**C**) The relationship between *lnc-BMP1-11* and *Cav-1* expression; (**D**) The expression of *lnc-BMP1-1* in types of lung cancer cells. RNA expression was analyzed using RT-PCR, the RNA levels were normalized against β-actin mRNA. Each bar represents the means ± SD of three independent experiments.

The clinical characteristics of the 276 patients with LC included in this study has been published in our previous studies conducted with the same subjects [39], another 17 patients were newly enrolled, the clinical characteristic of the study subjects are shown in Supplementary Table 2. As shown in Figure 1A, *lnc-BMP1-1* is down-regulated in lung cancer tissues, when comparing to their adjacent normal tissues (*P*=8.514×10^-7^). Therefore, we considered that *lnc-BMP1-1* is associated with lung cancer development and might exert tumor suppressor function.

The clinical data of these subjects are shown in Table 1. The smoking history is associated with the expression of *lnc-BMP1-1* (*P*=0.027). Patients with lower expression level of *lnc-BMP1-1* are more likely to be cigarette smokers or the ever ones, suggesting a possible association of *lnc-BMP1-1* and cigarette smoking-related genes. *Lnc-BMP1-1* is also associated with distant metastasis, though it isn’t a protection factor (*P*=0.002).

**Table 1:** The expression of *lnc-BMP1-1* and clinical characteristics of lung cancer patients.

**Clinical characteristics**	***lnc-BMP1-1* expression N(%)**		**Total Cases N(%)**	***P^a^***
	**Low**	**High**		
Age				
<60	139 (84.2)	26 (15.8)	165 (56.3)	0.499
≥60	104 (81.2)	24 (18.8)	128 (43.7)	
Gender				
Male	177 (85.5)	30 (14.5)	207 (70.6)	0.069
Female	66 (76.7)	20 (23.3)	86 (29.4)	
Smoking				
No	77 (76.2)	24 (23.8)	101 (34.5)	0.027
Yes	166 (86.5)	26 (13.5)	192 (65.5)	
Drinking				
No	185 (83.3)	37 (16.7)	222 (75.8)	0.749
Yes	58 (81.7)	13 (18.3)	71 (24.2)	
tumor history				
No	213 (83.5)	42 (16.5)	255 (87.0)	0.484
Yes	30 (78.9)	8 (21.1)	38 (13.0)	
Lc history				
No	228 (83.5)	45 (16.5)	273 (93.2)	0.354
Yes	15 (75.0)	5 (25.0)	20 (6.8)	
Clinical Stage				
Ⅰ+Ⅱ	93 (86.9)	14 (13.1)	107 (36.5)	0.170
Ⅲ+Ⅳ	150 (80.6)	36 (19.4)	186 (63.5)	
Histological subtype				
Adenocarcinoma	112 (82.4)	24 (17.6)	136 (46.4)	0.189
Squamous carcinoma	70 (79.5)	18 (20.5)	88 (30.0)	
Large cell carcinoma	8 (72.7)	3 (27.3)	11 (3.8)	
Small cell carcinoma	27 (96.4)	1 (3.6)	28 (9.6)	
Others	26 (86.7)	4 (13.3)	30 (10.2)	
T status				
1+2	126 (80.3)	31 (19.7)	157 (53.6)	0.190
3+4	117(86.0)	19 (14.0)	136 (46.4)	
N status				
0	91 (79.1)	24 (20.9)	115 (39.2)	0.164
1+2+3	152 (85.4)	26 (14.6)	178 (60.8)	
M status				
0	161 (78.5)	44 (21.5)	205 (70.0)	0.002
1	82 (93.2)	6 (6.8)	88 (30.0)	

**NOTE:** Comparisons between groups were analyzed as described in the Methods. Groups: High=patients with higher expression of *lnc-BMP1-1* in lung cancer tissues compared with their adjacent normal lung tissues; Low = patients with lower expression of *lnc-BMP1-1* in lung cancer tissues compared with their adjacent normal lung tissues. a, Pearson *χ*^2^ test for high vs. low gene (*lnc-BMP1-1* or *Cav-I*) expression groups combining clinical characteristics. Significance (bold values), p<0.05.

Abbreviations: N, number of subjects in each group; Lc history, lung cancer history.

### Effect of *lnc-BMP1-1* on cell proliferation and migration

According to the relationship between *SFTPC* and adenocarcinoma, with qrt-PCR detection (Figure 1D), we selected A549, GLC82 and PC9 adenocarcinoma cell types to further examine the *lnc-BMP1-1* function. Through observation with fluorescence microscope, we found that the transfection efficiency of *lnc-BMP1-1* in these three types of cells are above 80%; compared to that of the control cells, the fold change of *lnc-BMP1-1* expression in ‘A549-BMP’, ‘GLC82-BMP’, ‘PC9-BMP’ cells are about 700, 3000, 4000 times, respectively (Supplementary Figure 1).

**Figure 2:**
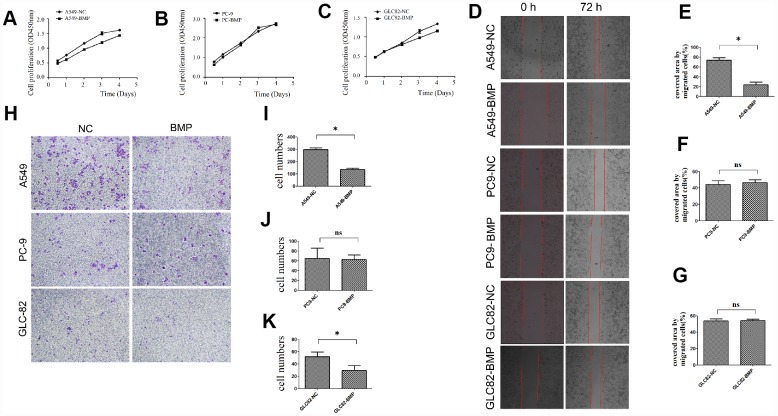
**The cell growth or migration of lung cancer cells with lnc-BMP1-1 over-expression vs. NC cells.** (**A**) The cell proliferation of A549-BMP vs. A549-NC, (**B**) PC9-BMP vs. PC9-NC, (**C**) GLC82-BMP vs. GLC82-NC, respectively; (**D**) Comparison of the wound widths (40x) of lung cancer cells with Inc-BMP1-1 over-expression vs. NC cells after 72 hrs, the relative bar graph are A549-BMP vs. A549-NC(E), PC9-BMP vs. PC9-NC(F), GLC82-BMP vs. GLC82-NC(G); the migration capacity of both A549-BMP and GLC82BMP cells were reduced; however, there's no significant difference of PC9-BMP vs. PC9-NC cells; with transwell migration experiments (40 x), the cell migration capacity of A549-BMP vs. A549-NC was proved to be decreased again (H), the relative bar graph was A549-BMP vs. A549-NC(I), PC9-BMP vs. PC9-NC(J), GLC82-BMP vs. GLC82NC (K), respectively. Data are represented as means ± SD. *P < 0.05, ns P> 0.05

Through detecting the cell viability, we found that the proliferation of A549-BMP cells was inhibited by the over-expressed *lnc-BMP1-1* when compared to A549-NC cells (Figure 2A), the proliferation of GLC82-BMP cells had also been inhibited when compared to GLC82-NC cells (Figure 2C); however, the inhibition on the proliferation of PC9-BMP cells is not obvious (Figure 2B).

With Cell Wound Scratch (Figure 2D) and Transwell (migration) assay (Figure 2H), we found that over-expression of *lnc-BMP1-1* could inhibit the migration of A549-BMP cells when compared to A549-NC cells. But no significant difference was observed in GLC82-BMP and PC9-BMP cells, compared to their GLC82-NC and PC9-NC cells, respectively.

### The Bcl-2 protein level is decreased by over-expressed *lnc-BMP1-1*

**Figure 3:**
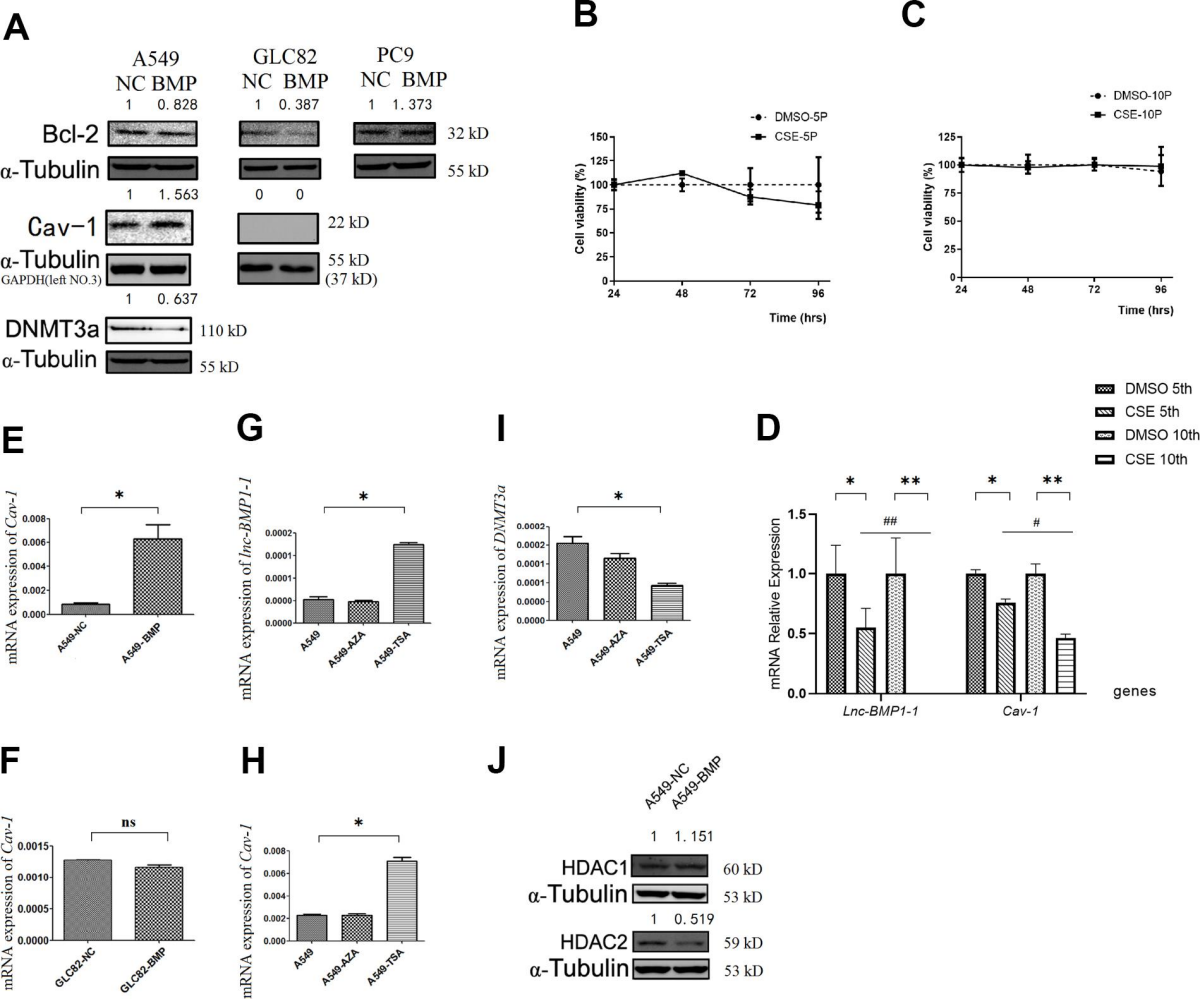
**The RNA or protein expression changes of lung cancer cells with *lnc-BMPl-1* over-expression vs. NC cells, the CSE vs. DMSO treated 16HBE cells, and 5-AzaC or TSA treatment.** (**A**) The protein expression of Bcl-2, Cav-1 and DNMT3a in different lung cancer cell lines with *lnc-BMP1-1* over-expression vs. NC cells; the protein expression were detected using western blotting analyses, protein expression was normalized against GAPDH or α-tubulin protein; the value above each band indicates the fold change of protein expression relative to their control; (**B**) The cell viability of CSE (vs. DMSO) treated 16HBE cells at 5^th^ passage; (**C**) The cell viability of CSE (vs. DMSO) treated 16HBE cells at 10^th^ passage; (**D**) The expression of *lnc-BMP1-1*, *Cav-1* were reduced in CSE treated 16HBE cells both in 5^th^ and 10^th^ passage, and made greater decline in 10^th^ than in 5^th^ passage (the same gene was compared in 5^th^ and 10^th^ generation of CSE treated 16HBE cells, *#P<*0.05, *##P<*0.01). The mRNA expression of *Cav-1* in A549-BMP vs. A549-NC (**E**), GLC82-BMP vs. GLC82-NC(F), respectively; In A549 cells treated with TSA, the expression of *lnc-BMP1-1* (**G**) and Cav-7 (**H**) were increased, while *DNMT3a* (**I**) was decreased; The protein expression of HDAC2 (**J**) was decreased in A549-BMP cells. Data are represented as means ± SD. *P< 0.05, **P<0.01.

Bcl-2 protein level is a most frequently used indicator for apoptosis resistance of cancer cells [40–42]. Compared to that of the A549-NC cells, the Bcl-2 protein level in A549-BMP cells is decreased by over-expressed *lnc-BMP1-1* (Figure 3A), indicating a decline of anti-apoptosis capacity of the A549-BMP cells, which also means that the over-expressed *lnc-BMP1-1* is helpful in improving the malignant phenotype of A549 cells, a result in accordance to the migration experiment results above. The protein level of Bcl-2 increased slightly in PC9-BMP cells, but no change was observed in the above migration experiment. We have thus concluded that *lnc-BMP1-1* has a different regulation mechanism in PC9 cells than in A549 and GLC-82 cells. Therefore, the PC9 cells are ruled out from our further experiments.

### *Cav-1* is probably the target gene of *lnc-BMP1-1*

To clarify how *lnc-BMP1-1* functions in lung cancer development, we first tried to find the downstream genes that are regulated by it. Based on the systematical analysis, three genes are taken into consideration, they are ‘ATP-binding cassette, sub-family A (*ABC1*), member 3 (*ABCA3*)’, ‘Caveolin-1 (*Cav-1*)’, and ‘natriuretic peptide receptor A (*NPR1*)’. Combining bio-information and literature analysis, *NPR1* and *Cav-1* were suggested as target genes regulated by *lnc-BMP1-1.* The expression of *NPR1* and *Cav-1* were detected in tissue samples by qrt-PCR.

Only the expression of *Cav-1* is significantly decreased in lung cancer population, and positively correlated with that of *lnc-BMP1-1* (R=0.211, *P* < 0.001) (Figure 1B and 1C). In 293 pairs of lung cancer tissues and their adjacent normal tissues (Table 2), *Cav-1* is also found to be associated with smoking (*P* = 0.011).

**Table 2:** The expression of *Cav-1* and clinical characteristics of lung cancer patients.

**Clinical characteristics**	***Cav-1* expression N(%)**		**Total Cases N(%)**	***P^a^***
	**Low**	**High**		
Age				
<60	133(80.6)	32(19.4)	165(56.3)	0.757
≥60	105(82.0)	23(18.0)	128(43.7)	
Gender				
Male	171(82.6)	36(17.4)	207(70.6)	0.348
Female	67(77.9)	19(22.1)	86(29.4)	
Smoking				
No	74(73.3)	27(26.7)	101(34.5)	0.011
Yes	164(85.4)	28(14.6)	192(65.5)	
Drinking				
No	180(81.1)	42(18.9)	222(75.8)	0.909
Yes	58(81.7)	13(18.3)	71(24.2)	
tumor history				
No	207(81.1)	48(18.9)	255(87.0)	0.953
Yes	31(81.6)	7 (18.4)	38(13.0)	
Lc history				
No	222(81.3)	51(18.7)	273(93.2)	0.775
Yes	16(80.0)	4(20.0)	20 (6.8)	
Clinical Stage				
Ⅰ+Ⅱ	87(81.3)	20(18.7)	107(36.5)	0.979
Ⅲ+Ⅳ	151(81.2)	35(18.8)	186(63.5)	
Histological subtype				
Adenocarcinoma	113(83.1)	23(16.9)	136(46.4)	0.149
Squamous carcinoma	72(81.8)	16(18.2)	88(30.0)	
Large cell carcinoma	6(54.5)	5(45.5)	11(3.8)	
Small cell carcinoma	21(75.0)	7(25.0)	28(9.6)	
Others	26(86.7)	4(13.3)	30(10.2)	
T status				
1+2	128(81.5)	29(18.5)	157(53.6)	0.887
3+4	110(80.9)	26(19.1)	136(46.4)	
N status				
0	93(80.9)	22(19.1)	115(39.2)	0.899
1+2+3	145(81.5)	33(18.5)	178(60.8)	
M status				
0	171(83.4)	34(16.6)	205(70.0)	0.144
1	67(76.1)	21(23.9)	88(30.0)	

NOTE: Comparisons between groups were analyzed as described in the Methods. Groups: High=patients with higher expression of *Cav-1* in lung cancer tissues compared with their adjacent normal lung tissues; Low = patients with lower expression of *Cav-1* in lung cancer tissues compared with their adjacent normal lung tissues. a, Pearson *χ*^2^ test. Significance (bold values), p<0.05.

Abbreviations: N, number of subjects in each group; Lc history, lung cancer history.

### The expression of *lnc-BMP1-1* and *Cav-1* in 16HBE cells treated with cigarette smoke extract (CSE)

The expression of *lnc-BMP1-1* in 16HBE cells treated with cigarette smoke extract (CSE) was shown, including the 5^th^ and 10^th^ generations of 16HBE cells. As to Figure 3B and 3C, be it DMSO or CSE treatment, the cell viability alteration was not obvious. In comparison to that in the control cells (DMSO treated only), the *lnc-BMP1-1* expression in 5^th^ and 10^th^ generation cells is decreased, indicating the sensitivity of *lnc-BMP1-1* to cigarette smoke. Notably, the expression of *lnc-BMP1-1* expression in 10^th^ generation is undetectable. Meanwhile, the *Cav-1* expression is also decreased along with the decreased of *lnc-BMP1-1* expression. Both the expression of *lnc-BMP1-1* and *Cav-1* showed greater decline in the 10^th^ than in the 5^th^ generation, these results further reinforce our viewpoint that the expression of *lnc-BMP1-1* and *Cav-1* in human bronchial epithelial cells might be decreased because of the exposure to cigarette smoking (Figure 3D).

### The mRNA and protein level of *Cav-1* are increased by over-expressed *lnc-BMP1-1*

When compared A549-BMP to A549-NC cells, both the mRNA and protein level of *Cav-1* are increased as a result of the over-expression of *lnc-BMP1-1* (Figure 3E and 3A). Meanwhile, no alteration of *Cav-1* was observed in GLC-82 cell (Figure 3F and 3A), both on the mRNA and the protein level. It is possible that *Cav-1* is not regulated by *lnc-BMP1-1* in the GLC-82 cell line*.* Therefore, we select the A549 cell line to further explore the transcription impact that *lnc-BMP1-1* has on *Cav-1.*

### The tumor growth in nude mice of A549 cells is inhibited by the over-expression of *lnc-BMP1-1*

**Figure 4:**
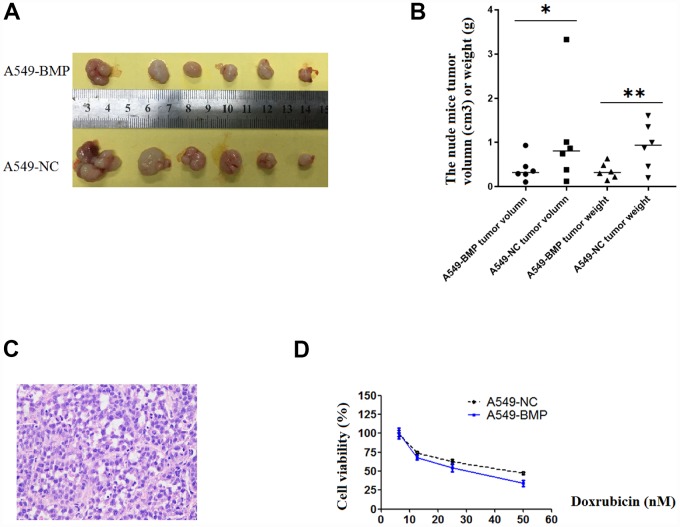
**The tumor growth in nude mice and the results of anti-cancer drug trial in A549-BMP vs. A549-NC cells.** (**A**) Tumor of A549-BMP vs A549-NC cells; (**B**) Tumor volume and tumor weight; (**C**) The HE staining of nude mice tumor tissues; (**D**) The drug sensitivity to doxorubicin hydrochloride was enhanced in A549-BMP vs A549-NC cells. Data are represented as means ± SD. *P< 0.05, **P<0.01.

21 days after injection, the tumor in nude mice injected with A549-NC cells (A549-NC nude mice) had grown larger in size than that in nude mice injected with A549-BMP cells (A549-BMP nude mice). At day 28 (Figure 4A), the average weight of tumors in the A549-BMP nude mice group is 0.35g ± 0.17g, while it is 0.48g ± 0.55g in the A549-NC nude mice group; the average size of tumors in the A549-BMP nude mice group is 0.40 ± 0.28 cm^3^, while it is 1.07 ± 1.15 cm^3^ in theA549-NC nude mice group (Figure 4B). The represented photo of H & E staining of the tumor tissues of nude mice is shown in Figure 4C. The above data show the inhibition of tumor growth by the over-expression of *lnc-BMP1-1* in subcutaneous tumorigenesis of nude mice.

### Explore the mechanism of *lnc-BMP1-1* in regulating *Cav-1*

To understand in which epigenetic regulatory manner *lnc-BMP1-1* exerts its function and what are the relevant molecules, A549 cells were treated with 5-AzaC and TSA, respectively. As a result the expression of *lnc-BMP1-1* (Figure 3G), *Cav-1* mRNA (Figure 3H) was increased, while that of *DNMT3a* was decreased (Figure 3I), but not that of *DNMT3b* and *DNMT1* (supplementary Figure 2A and 2B). As shown in Figure 3G–3I, we found that histo-acetylation modification might be the dominant mechanism through which *lnc-BMP1-1* exerts function. In response to the TSA treatment, the expression of *lnc-BMP1-1* and *Cav-1* are increased significantly. We’ve carried out the detection of the methylation status of the promoter of *Cav-1*, and no difference was found between that in A549-BMP and in A549-NC cells (supplementary Figure 2C), even though there is obvious change in DNMT3a protein expression (Figure 3A). The above results again proved that histo-acetylation regulation pathway is more dominant than the DNA methylation regulation.

As to Figure3J, the HDAC2 protein is decreased in A549-BMP cells, which might be responsible for the transcription promotion of *Cav-1* by *lnc-BMP1-1.* We have previously found that TSA, an HDACIs, could increase the expression of both *lnc-BMP1-1* and *Cav-1* (Figure 3G and 3H), indicating a possible interaction of *lnc-BMP1-1* and HDAC2. To some degree, we might conclude that the inhibited transcription of *Cav-1* resulted from decreased histo-acetylation level caused by decrease of *lnc-BMP1-1.*

### The enhancement of the drug sensitivity of Doxorubicin hydrochloride (DOX) by over-expressed *lnc-BMP1-1* in A549 cells

As to Figure 4D, at the concentration of 50nM of Doxorubicin hydrochloride (DOX), the cell viability was 37% of A549-BMP cells when compared to 50% of A549-NC cells. Over-expressed *lnc-BMP1-1* markedly enhanced the drug sensitivity of Dox to A549 cells. However, there’s no improvement in sensitivity to Cisplatin of A549 cells resulting from the over-expression of *lnc-BMP1-1*(Supplementary Figure 2D).

## DISCUSSION

*Sftpc* is specifically expressed in lung tissue, according to NCBI database. Moreover, *lnc-BMP1-1*, being transcribed from the intron area of *Sftpc*, is also rich in lung tissues base on our detection. There are promising biomarkers for lung cancer diagnosis, such as *TTF1* (thyroid transcription factor 1), folate receptor-positive circulating tumor cells [43, 44], but no lncRNAs are ready for broad clinical application [45], which calls for more exploration into the function and mechanism of lncRNAs. It is widely accepted that the expression of lncRNAs are tissue-specific, suggesting that *lnc-BMP1-1* is a potential biomarker for lung cancer screening.

Smokers or former smokers are more likely to have lower level of *lnc-BMP1-1* expression. We have proven that the expression of both *lnc-BMP1-1* and *Cav-1* could be reduced by CSE, further supporting our hypothesis that *lnc-BMP1-1* is involved in the pathway of cigarette smoking-induced lung cancer. Abnormal expression of lncRNAs will cause lung cancer, breast cancer and other diseases, resulting from the alternation in conformation of molecular combination and gene regulation, including DNA methylation [46], transcription, phosphorylation, acetylation, ubiquitination [47] and so on. For example, if the transcription of a tumor suppressor gene *FENDRR*, functioning as a competing endogenous RNA to compete binding site with miR-424 towards *FOXF1*, is inhibited, the miR-424 will depress the translation of *FOXF1 by* sequence matching to 3’ UTR of its mRNA, resulting in increased lung cancer risk [48]. In our study, we have revealed that *lnc-BMP1-1* might promote the transcription of *Cav-1* through down-regulating the expression of HDAC2, which is a post-translational regulation. It is reported that HDAC enzymes frequently over-express in cancer patients [49] and HDACs are important therapeutic targets. As to the low toxic of HDAC inhibitors (HDACIs) to normal cells, some HDACIs, such as SAHA [50], Farydak (panobinostat) [51] have been approved by the US Food and Drug Administration. Notably, as a type of post-translational regulation, histo-acetylation dominant regulatory mechanism is thought to provide better prognosis than DNA methylation dominant mechanism in cancer patients [52]. In the present study, the HDAC2 protein level had been found to decrease as a result of over-expressing *lnc-BMP1-1*, indicating an increased histo-acetylation modification level, which we consider as the reason that accounts for promoted *Cav-1* transcription; therefore, *lnc-BMP1-1* is a possible endogenous HDAC inhibitor.

In addition, probably there are more than one regulatory mechanism during carcinogenesis and cancer development. Comparable to our conclusion, other researches have proven that combining HDACIs and DNMTIs treatment might offer greater potential in cancer therapy, instead of treatment with either HDACIs or DNMTIs alone [53]. LncRNAs might interact with DNMTs to modify tumor suppressors [54]. Such as a study that have revealed the hypermethylation modification of DNA promoter of *Cav-1* is associated with the transcription inhibition of *Cav-1* in small cell lung cancer (SCLC) cells alone, and not with the transcription inhibition in non-small cell lung cancer (NSCLC) cells, a process which happens in a phosphorylation manner instead [55]. In our study, up-regulating the expression of *lnc-BMP1-1* in A549 cells did not show significant influence on the DNA methylation status of *Cav-1* promoter, even though there is down-regulation of DNMT3a at protein levels by *lnc-BMP1-1*. The above results indicate that *lnc-BMP1-1* has selected both DNA methylation and histo-acetylation modification to exert function, and *Cav-1* might be one of the target genes.

*Cav-1* is a gene characterized as related with cigarette smoke, oxidative stress, cisplatin sensitivity, as well as lung cancer development. Cav-1 attenuates hydrogen peroxide-induced oxidative damage to lung carcinoma cells [56]. Oxidative stress induced by cigarette smoke (CS) is considered a cause for lung tumorigenesis [19, 57–60], in which reactive oxygen species (ROS) are important mediators. Several drugs targeting ROS are under various stages of clinical development [24, 61]. LncRNAs alter the expression of generator and effector systems of redox regulation in a complex manner [54]. Recently, ROS was reviewed to alter chromatin structure and metabolism that impact the epigenetic landscape in cancer cells [61].

Clinical findings have revealed that once the TKI-sensitive patients begin to smoke, drug resistance will appear, in which course the membrane protein Cav-1 plays important roles [60]. Because *Cav-1* may be critical for albumin uptake in tumors and perhaps determine how the patients respond to albumin bound drug, such as cisplatin [25, 62]; it is also verified that over-expressed *Cav-1* enhanced the sensitivity to nab-paclitaxel in cancer cell lines and mouse xenograft models [28]. In the present study, over-expressed *lnc-BMP1-1* could increase the expression of *Cav-1* and enhance the sensitivity of A549 cells to Doxorubicin, a conclusion that sheds light on the usage of *lnc-BMP1-1* in cancer treatment.

There are limitations in the present study that *Cav-1* might not the sole target of *lnc-BMP1-1*, though the former is proven to be regulated by the latter. Similarly, it’s reported that high *Cav-1* expression also indicates a better overall survival (OS) for lung adenocarcinoma but not for squamous cell lung carcinoma [20]. The mechanism for the down-regulation of *lnc-BMP1-1* in lung cancer tissue might be caused by oxidative stress, but it remains elusive. It is anticipated that *lnc-BMP1-1* may not only be a diagnosis biomarker for lung tumor, but also reflect the complex biological courses of cancer development. In addition, the protein expression of *Bcl-2* is decreased as a result of *lnc-BMP1-1* over-expression, which is the focus in our future study.

## MATERIALS AND METHODS

### Study subjects

All study subjects selected in this study were Han Chinese originated from Southern or Eastern China. A total of 293 samples of cancer tissues and paired normal tissues were collected in this study. Among the 293 samples, 199 pairs of samples were collected between 2008 and 2015 from the Cancer Hospital Affiliated with Guangzhou Medical University, and1^st^ Affiliated Hospital and Cancer Hospital Affiliated with Kunming University, and 94 pairs of samples were collected between 2007 and 2016 from the 1^st^ Affiliated Hospital of Soochow University. The patients in the study have no genetic connections with one another. The study was approved by the Research Ethics Committee of Guangzhou Medical University (No. GMU201481473040) and followed clinical research guidelines. Written informed consents were obtained from all patients who participated in this study.

### qRT-PCR

Total cellular RNA was isolated with TRIzol (Invitrogen, Carlsbad, CA, USA) according to the manufacturer’s instructions. To detect the RNA expression of related genes, we first synthesised the strand cDNA using the RevertAidTM First Strand cDNA Synthesis Kit (Thermo Scientific, Carlsbad, CA, USA). Next, qRT-PCR was performed using the FastStart Universal SYBR Green Master kit (Roche, Mannheim, Germany) on an ABI7900HT PCR instrument with specific primers (shown in the Supplementary Table 1). All primers were synthesised by Invitrogen Ltd. and listed in Supplementary Table 1. β-actin was used as an internal control. The delta-delta CT method was used to quantify gene expression using the 7900 System SDS Software according to the recommended protocol.

### Cell culture

The human lung cancer cell lines (A549, GCL82, and PC9) and a normal bronchial epithelial cell line (16HBE) were purchased from the Cell Bank of Type Culture Collection of the Chinese Academy of Science, Shanghai Institute of Cell Biology. The A549, GCL82, and PC9 cell lines were cultured in RPMI-1640 medium (Gibco, life technologies, California, USA) with 10% fetal bovine serum (FBS) and penicillin (100UI/ mL)/streptomycin (100mg/mL) in standard conditions with 5% carbon dioxide at 37.

### Cigarette smoke extract (CSE) preparation and acute exposure

Commercial normal cigarettes (Double Happiness, Guangzhou Tobacco, Guangzhou, China) yield 15mg of tar and 1.4mg of nicotine under a standard smoking regimen. Cigarette burning-produced puffs were collected using an atmosphere sampler with a glass accessory containing 100% Dimethyl sulfoxide (DMSO, AR), then purified water was added into the compound, forming a 2% diluent, termed as cigarette smoke extract (CSE). 16HBE cells were treated with 10mL CSE for 24h and made a rest for another 24h; the cells grown every 3 days are considered one generation. The 5^th^ and 10^th^ generation cells were employed for the next detection.

### Cell transfection

To understand the function of *lnc-BMP1-1* in lung cancer, the human lung cancer adenocarcinoma cell lines A549, GLC82 and PC9 were transfected with over-expressed *lnc-BMP1-1* lentivirus vectors. The cDNA sequence of *lnc-BMP1-1* was obtained from Lncipedia database (version4.0), the coding fragment of *lnc-BMP1-1* was synthesised by PCR and then inserted into the pLVX-shRNA1 vector (Clontech Corporation, Tokyo, Japan) using the BamH I and EcoR I restriction sites.

The over-expression of *lnc-BMP1-1* in lung adenocarcinoma cell line A549, GLC82 and PC9 were denoted as ‘A549-BMP’, ‘GLC82-BMP’, ‘PC9-BMP’ and their corresponding empty vector control cells were denoted as ‘A549-NC’, ‘GLC82-NC’, ‘PC9-NC’ in the following text.

### Cell proliferation

Cell growth was measured by cell counting with the CCK-8 cell proliferation assay kit (Dojindo, Tokyo, Japan). In a 96-well plate, 1000 cells in 100 μL serum-free RPMI-1640 medium were plated per well. The plates were incubated at 37°C for 24 h, and 10 μL CCK-8 was added to each well. The cells were cultured for an additional 4 h at 37°C. With a reference wavelength of ‘650 nm’, the absorbance wavelength of the sample was 450 nm.

### Cell migration (Wound scratch and Trans-well migration)

Wound scratch method of the lentivirus-vectors-transfected A549, GLC82 and PC-9 cell lines were carried out to understand the contribution of *lnc-BMP1-1* to the cell migration capacity. Transfected cells were seeded on 24-well plates and then grown to confluence. The confluent monolayers were scratched with a pipette tip and maintained under certain conditions according to the protocol. Plates were washed once with fresh medium to remove non-adherent cells and then photographed. The percentage of open spaces covered by migrated cells was calculated with ImageJ software.

Trans-well migration assay of the lentivirus-vectors-transfected A549, GLC82 and PC-9 lung adenocarcinoma cell lines was conducted in an 8μm Transwell chamber (Costar, Corning Incorporated, NY). Cells grew to near confluence in a 5×5 cm culture vessel and then were placed in serum-free RPMI-1640 medium (Gibco) for 24 h. Cells were trypsinized and re-suspended in serum-free RPMI-1640 medium with 0.1% BSA. We seeded 2×10^4^ cells in the chamber, which was put in a 24-well plate containing RPMI-1640 with 20% FBS (Gibco). After 48 h of culture, non-migrating cells in the top section of the chambers were removed by swabbing. Migrated cells were counted manually in 10 random fields and their numbers were averaged.

### Xenograft tumor formation assay in nude mice

With A549-BMP and A549-NC cells, nude mice tumor transplantation experiments were employed to study the influence of *lnc-BMP1-1* on tumor growth inhibition in vivo. Five-week-old BALB/c nude mice were provided by Guangdong Medical Animal experiment Centre (Foshan, China). Transformed and control cells were washed twice and suspended with serum-free RPMI-1640 medium. Then, 5×10^6^ cells were resuspended in culture medium and injected subcutaneously into the neck pads of nude mice. The mice were housed in a pathogen-free environment and monitored every 2 days for tumor formation. All mice were sacrificed after 28 days, and the tumors were removed, weighed and fixed in 10% buffered formalin for pathological examination. This study was approved by the Ethics Committee of Guangzhou Medical University (NO. GZYDW201503512).

### 5-AzaC and TSA treatment

DNA methylation and histo-acetylation modification are two important mechanisms in epigenetic regulatory. To clarify through which epigenetical regulation mechanism *lnc-BMP1-1* functions on *Cav-1*, A549 cells were treated with 5-AzaC (5 μmol/L) or TSA (100 nmol/L) for 24 h, respectively. Then the expression of *lnc-BMP1-1*, *Cav-1*, and DNMTs were compared with that of untreated cells.

5-AzaC (Sigma) was dissolved in DMSO and stored at -20°C. TSA (Sigma) was dissolved in absolute ethanol at a concentration of 4 mM, shielded from light and stored at -20°C. Exponentially grown cells were treated with 5-AzaC (5 μmol/L) or TSA (100 nmol/L) for 24 h.

### Methyl-specific PCR (MSP)

MSP was carried out to analyze the DNA methylation level of *Cav-1* promoter. Methylation-specific PCR (MSP) was performed as previously described [37]. Briefly, genomic DNA was extracted as recommended by the EZNA-DNA kit (Omega). Next, 1 μg of purified DNA was subjected to bisulfite modification using a CpGenome DNA Modification Kit (Chemicon International). The bisulfite-treated DNA was stored at -80 until further used, MSP performed was performed using bisulfite-treated DNA, methylation primers and non-methylation primers. The primer sets used in this study were as follows: 5′-TTATTTCGAAGCGTTTGGGAG-3′ and 5′-AACACTCGTTTACATCTAATCG-3′ for the methylated reaction, and 5′-TTATTTTGAAGTGTTTGGGAG-3′ and 5′-AACACTCATTTACATCTAATCA-3′ for the unmethylated reaction [55].

### Western blotting

Total protein was extracted with cell lysis buffer (Cell Signaling Technology, Beverly, MA, USA) according to the manufacturer’s instructions, and the protein concentration was determined using the Bicinchoninic Acid (BCA) assay kit (Kangwei technology, Beijing, China). Western blotting analyses were performed as previously described [38]. Glyceraldehyde 3-phosphate dehydrogenase (GAPDH), α-tubulin primary antibodies were purchased from Cell Signaling Technology (Beverly, MA, USA); Cav-1, DNMT3a and Bcl-2 primary antibodies were purchased from Abcam (Cambridge, MA, USA). The horseradish peroxidase (HRP)-labelled goat anti-mouse and goat anti-rabbit IgG secondary antibodies were purchased from Santa Cruz Biotechnology (Santa Cruz, CA, USA).

### Hematoxylin and eosin staining of tumor tissues of nude mice

The tumor or adjacent normal tissues of nude mice were stained with Harris’ hematoxylin solution for 6 h at a temperature of 60~70 °C and were then rinsed in tap water until the water was colorless. Then 10% acetic acid and 85% ethanol in water were used to differentiate the tissue 2 times for 2 h and 10 h, respectively, and the tissues were rinsed with tap water. In the bluing step, we soaked the tissue in saturated lithium carbonate solution for 12 h and then rinsed it with tap water. Finally, staining was performed with eosin Y ethanol solution for 48 h.

### Anti-cancer drug sensitivity

To understand whether *lnc-BMP1-1* could enhance the sensitivity of A549 cells to anti-cancer drug, including Cisplatin (DDP, 200~600μg/mL) and Doxorubicin hydrochloride (DOX, 10~50 nM). An observation was made every 24h and cell viability was checked using CCK8 kit at the end point of drug treatment.

### Statistical analysis

All tests were finished with SPSS19.0. Paired t-test was used to analyze the different expression levels of genes in lung cancer tissues and adjacent non-cancerous tissues. Linear regression models were used to analyze the correlation between *lnc-BMP1-1* and *Cav-1*. Chi-square test and Logistic regression model were used to analyze the expression of *lnc-BMP1-1* and clinical characteristics of lung cancer patients. Independent t-test was used to analyze the cell migration capability. Repetitive measurement deviation analysis was used to analyze the difference in cell proliferation. All statistical tests were two sides, *P* < 0.05 was considered statistically significant.

## Supplementary Material

Supplementary Figures

Supplementary Tables
