# Supplementary material for: Down expression of lnc-BMP1-1 decreases that of Caveolin-1 is associated with the lung cancer susceptibility and cigarette smoking history
Source: Aging (Albany NY). 2020 Jan 4;12(1):462–80. doi: 10.18632/aging.102633 (PMC6977698; doi:10.18632/aging.102633)
Supplement: Supplementary Figures [file aging-12-102633-s002..pdf]

SUPPLEMENTARY FIGURES

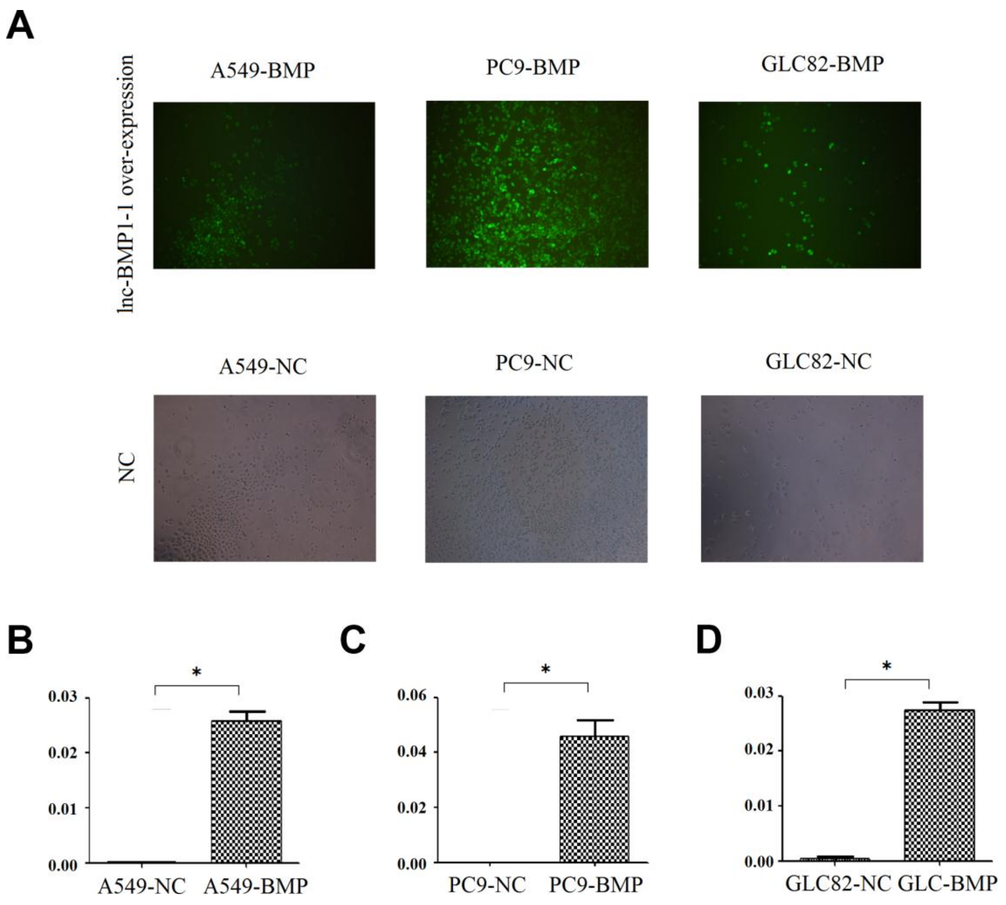

Supplementary Figure 1. *Lnc-BMPl-1* was over-expressed in lung cancer cells. (**A**) The represented fluorescence microscope pictures of A549, PC9 and GLC82 cells with *lnc-BMP1-1* over-expression, and the corresponding bar graph of (**B**) A549-BMP vs A549-NC cells; (**C**) PC9-BMP vs PC9-NC cells; (**D**) GLC82-BMP vs. GLC82-NC cells. Data are represented as means±SD. *P<0.05, **P<0.01.

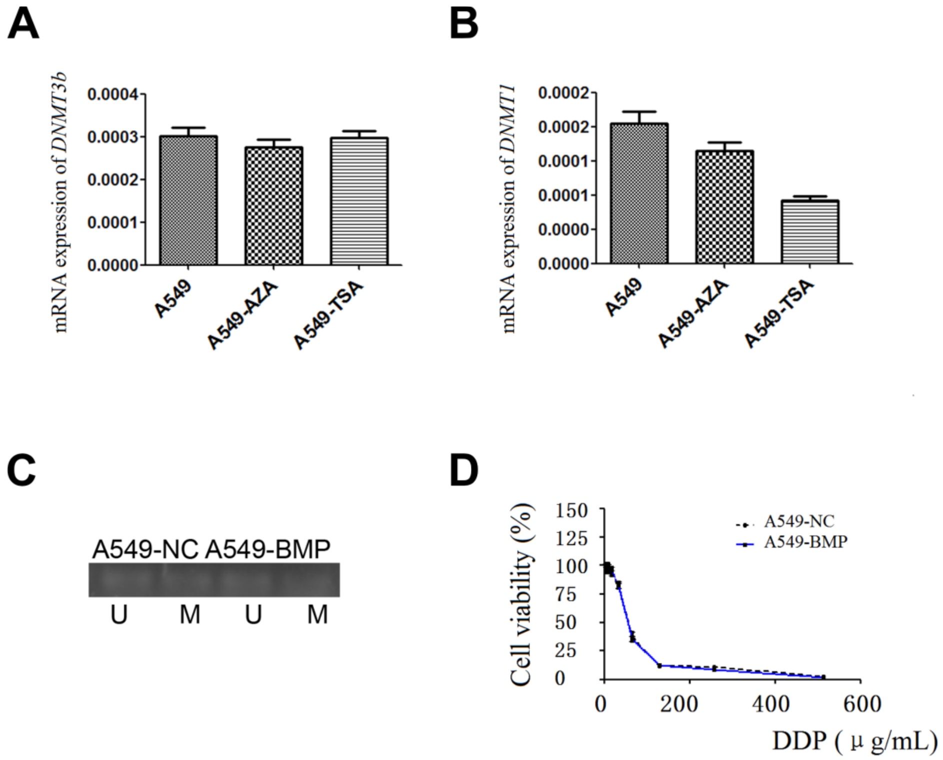

Supplementary Figure2. The expression of *DNMTs* with 5-AzaC and TSA treatment, the methylation status of *Cav-1* promoter in A549-BMP vs. A549-NC cells, and the drug sensitivity trial of DDP. (**A**) The expression of *DNMT3b*mRNA of A549 cells with 5-AzaC or TSA treatment; (**B**) The expression of *DNMT1* mRNA of A549 cells with 5-AzaC or TSA treatment; (**C**) There's no significant difference between A549-BMP and A549-NC cells in the DNA methylation status of *Cav-1* promoter; (**D**) There's no significant difference between A549-BMP and A549-NC cells in the cell viability under the same treatment of DDP. Data are represented as means ± SD.
