# Supplementary material for: Down expression of lnc-BMP1-1 decreases that of Caveolin-1 is associated with the lung cancer susceptibility and cigarette smoking history
Source: Aging (Albany NY). 2020 Jan 4;12(1):462–80. doi: 10.18632/aging.102633 (PMC6977698; doi:10.18632/aging.102633)
Supplement: Supplementary Tables [file aging-12-102633-s001..pdf]

SUPPLEMENTARY TABLES

Supplementary Table 1. The primers used in this study.

Gene name	Forward primers(5′-3′)	Reverse primers(5′-3′)
lnc-BMP1-1	TTCTGGAGATGAGCATTGGGG	AGTGGAGCCGATGGAGAAGGT
DNMT1	TTGGAGAACGGTGCTCATGCTTA	CATCTGCCATTCCCACTCTACGG
DNMT3a	GGCAAACTGAGGTAGCGACA	AAAAAAAGGGAAGGGGGAG
DNMT3b	AAGAGTTGGGCATAAAGGTAGG	GCTGGATTCACATTTGAGAGAT
Cav-1	GCAGCCTCCCTGAAGACCAAA	TGCCAGATGAAGCCCAGAAGT
ABCA3	TCTTCGAGCACCCCTTCAAC	GTAGTGTGCCAGCCTTCTGT
NPR1	TTGCTCGGCATTCTGATTGTCTC	CCTCTCAAGGCTACTGGGCTCAAC
β-actin	GGCGGCACCACCATGTACCCT	AGGGGCCGGACTCGTCATACT
GAPDH	GGAGTCAACGGATTTGGTCGTATTG	TCTCGCTCCTGGAAGATGGTGAT

Supplementary Table 2. Demographics and clinical characteristics of studied samples.

Clinical Characteristics		Southern Samples N (%)	Eastern Samples N (%)	Pearson χ2	P value
Total		199(67.9)	94(32.1)		
Age(years)				0.325	0.569
	< 60	115(57.8)	51(54.3)		
	≥60	84(42.2)	43(45.7)		
Sex				0.013	0.910
	Female	58(29.1)	28(29.8)		
	Male	141(70.9)	66(70.2)		
Family history of cancer				0.666	0.414
	No	176(88.4)	84(89.4)		
	Yes	23(11.6)	10 (10.6)		
Smoking				0.512	0.400
	No	72(36.2)	30(31.9)		
	Yes	127(63.8)	64(68.1)		
Drinking				0.183	0.270
	No	150(75.4)	73(77.7)		
	Yes	49(24.6)	21(22.3)		
Clinical Stage				2.794	0.192
	I+II	77(38.7)	29(30.9)		
	III+IV	122(61.3)	65(69.1)		
Histological types				1.842	0.771
	Adenocarcinoma	95(47.7)	41(43.6)		
	Squamous cell carcinoma	56(28.1)	31(33.0)		
	Large cell carcinoma	6(3.0)	5(5.3)		
	Small cell lung cancer	21(10.6)	8(8.5)		
	Other carcinomas*	21(10.6)	9(9.6)		

*Mixed-cell or undifferentiated carcinoma
